# Comparison of acute myeloid leukemia and myelodysplastic syndromes with *TP53* aberrations

**DOI:** 10.1007/s00277-022-04766-2

**Published:** 2022-01-26

**Authors:** Sayantanee Dutta, Jennifer Moritz, Gudrun Pregartner, Gerhard G. Thallinger, Ilona Brandstätter, Karin Lind, Simin Rezania, Freya Lyssy, Andreas Reinisch, Armin Zebisch, Andrea Berghold, Albert Wölfler, Heinz Sill

**Affiliations:** 1 Division of Hematology, Medical University of Graz, Auenbruggerplatz 38, 8036 Graz, Austria; 2 Institute for Medical Informatics, Statistics and Documentation, Medical University of Graz, Graz, Austria; 3 Institute of Biomedical Informatics, Graz University of Technology, Graz, Austria; 4 BioTechMed-Graz, Graz, Austria; 5 Department of Blood Group Serology and Transfusion Medicine, Medical University of Graz, Graz, Austria; 6 Otto-Loewi-Research Center for Vascular Biology, Immunology and Inflammation, Division of Pharmacology, Medical University of Graz, Graz, Austria

**Keywords:** *TP53*, Acute myeloid leukemia, Myelodysplastic syndromes, Stem cell disorder, EAp53 and RFS score

## Abstract

*TP53* aberrations are found in approximately 10% of patients with acute myeloid leukemia (AML) and myelodysplastic syndromes (MDS) and are considered early driver events affecting leukemia stem cells. In this study, we compared features of a total of 84 patients with these disorders seen at a tertiary cancer center. Clinical and cytogenetic characteristics as well as immunophenotypes of immature blast cells were similar between AML and MDS patients. Median overall survival (OS) was 226 days (95% confidence interval [CI], 131–300) for the entire cohort with an estimated 3-year OS rate of 11% (95% CI, 6–22). OS showed a significant difference between MDS (median, 345 days; 95% CI, 235–590) and AML patients (median, 91 days; 95% CI, 64–226) which is likely due to a different co-mutational pattern as revealed by next-generation sequencing. Transformation of *TP53* aberrant MDS occurred in 60.5% of cases and substantially reduced their survival probability. Cox regression analysis revealed treatment class and *TP53* variant allele frequency as prognostically relevant parameters but not the *TP53*-specific prognostic scores EAp53 and RFS. These data emphasize similarities between *TP53* aberrant AML and MDS and support previous notions that they should be classified and treated as a distinct disorder.

**Supplementary Information:**

The online version contains supplementary material available at 10.1007/s00277-022-04766-2.

## Introduction

The *TP53* gene, located at chromosome 17p13.1, encodes p53—a fundamental tumor suppressor highly conserved during evolution. Among a multitude of different physiological functions, p53 is activated by several extrinsic and intrinsic stress signals including DNA damage, oncogene activation, hypoxia, and nutrient deprivation. Dependent on the activation signal, p53 induces a multitude of downstream signals aimed at sustaining cellular homeostasis. Importantly, by pursuing these functions, p53 acts in a cell context–specific manner. There is tight regulation of p53 at the transcriptional, post-transcriptional, and post-translational levels, respectively, conferring fine-tuning of this essential cellular protein [1, 2].

p53 displays pivotal functions in normal hematopoietic stem and progenitor cells being involved in their proliferation, differentiation, and apoptosis [3]. Using transgenic mice, it could be shown that p53 conveys quiescence during steady-state hematopoiesis [4]. Murine p53-deficient stem cells showed enhanced self-renewal with increased serial replating and repopulating capacity in vitro and in vivo, respectively. In cooperation with oncogenic mutations like Kras^G12D^, p53 loss led to indefinite self-renewal of these cells with a propensity for transformation into leukemia-initiating cells [5].

Acute myeloid leukemia (AML) is an aggressive hematopoietic malignancy derived from transformed HSPCs ultimately leading to bone marrow failure. Myelodysplastic syndromes (MDS) are clonal bone marrow disorders characterized by ineffective hematopoiesis and peripheral blood cytopenias progressing to AML in a substantial number of cases. Both disease entities are highly heterogeneous with respect to biological and clinical features [6, 7]. In AML and MDS, *TP53* aberrations are constantly observed at a frequency of approximately 10% with steep increases in older patients and those with therapy-related myeloid neoplasms. Predominantly, they constitute missense mutations located in the DNA binding domain of the gene. However, other types of mutations—nonsense variants, small insertions and deletions—as well as chromosomal losses encompassing the *TP53* locus and combined aberrations are also observed [8, 9]. These aberrations may lead to loss of physiological functions, exert a dominant-negative phenotype on wild-type p53, or—in some instances—result in a gain of novel, oncogenic properties. Concerning their origin, *TP53* mutations are somatically acquired in the majority of patients affecting hematopoietic stem and progenitor cells; however, germline mutations characterizing the Li-Fraumeni (LF) and LF-like syndromes are also observed in these disorders [10–15]. Recently, it was reported that individuals with clonal hematopoiesis of indeterminate potential with *TP53* mutations exhibit a high risk of progression to AML [16]. Patients with AML and MDS with *TP53* aberrations usually have a dismal prognosis even when treated with intensive regimens including allogeneic hematopoietic stem cell transplantation (HSCT) [17, 18].

Based on biological and clinical features, we recently proposed that AML and MDS with *TP53* aberrations should be regarded as a distinct disease entity [19]. In this work, we compare data from such patient cohorts referred to a tertiary hematology center to corroborate our proposition.

## Methods

The study cohort consisted of adult patients suffering from AML and MDS with *TP53* aberrations, seen at our institution—a tertiary hematology center for a population of approximately 1.5 million people—between November 2014 and June 2021. Only those patients whose *TP53* aberrations were detected at diagnosis were included. Diagnosis and risk stratification of myeloid neoplasms were performed according to standard criteria including conventional karyotyping as well as fluorescent in situ hybridization (FISH). For molecular studies, genomic DNA extracted from bone marrow biopsies was analyzed for mutations in up to 44 myeloid-associated genes using an Ion Torrent next-generation sequencing (NGS) platform (Thermo Fisher Scientific) as described previously [20]. With respect to *TP53*, all coding exons and flanking exon–intron boundaries were sequenced with a lower limit of detection set at 5% mutant allele reads. Variants were classified according to the VarSome (https://varsome.com) and *TP53* databases (https://p53.iacr.fr), and only pathogenic and likely pathogenic ones were included. Treatment categories comprised intensive chemotherapy and allogeneic hematopoietic stem cell transplantation (HSCT), non-intensive therapies (lenalidomide, hypomethylating agents, low-dose cytarabine, and growth factors), and best supportive care including hydroxyurea. All patient data were retrieved from openMEDOCS, a regional hospital–based documentation system.

### Statistical analysis

To compare clinical and biological parameters, the Mann–Whitney *U* and Fisher’s exact tests were used for continuous and categorical parameters, respectively, and median (range) or numbers (*n*, in %) are used to descriptively summarize the data. Kaplan–Meier analysis and the log-rank test were used to describe and compare overall survival (OS), calculated as time from diagnosis to either death or last-follow up, and the median OS with 95% confidence intervals (CI) is presented. To identify relevant prognostic parameters, Cox regression analyses were performed taking the particular disease (AML, MDS), age, sex, the *TP53* variant allele frequency (VAF), *TP53*-specific scores, and the treatment class into account. The *TP53* scores were explored as previously described by our group [21]. Briefly, we initially assessed the impact of the location and consecutive amino acid alteration of a particular *TP53* variant. Then, we investigated the evolutionary action score (EAp53) focusing on the evolutionary sensitivity to sequence variation and amino acid conservation of missense mutations. Finally, the relative fitness score (RFS) based on in vitro growth properties of particular *TP53* variants was evaluated. For the multivariable analysis, all parameters with *p* < 0.05 in the univariable analysis as well as age and sex were considered. The analyses were performed using R version 4.1.0 (https://www.R-project.org/).

### Immunophenotyping

The immunophenotype of blast cells was compared between AML and MDS patients showing *TP53* aberrations. Multicolor flow cytometry was performed using a Navios cytometer (Beckman Coulter, USA) with harmonized baseline settings as described previously [22]. After erythrocyte lysis, peripheral blood (PB) or bone marrow (BM) cells were washed twice with D-PBS (Life Technologies) and stained with the appropriate antibodies (Supplementary Table 1) for 18 min in the dark. At least 30,000 events were recorded and data were analyzed using Kaluza software (Beckman Coulter). Blasts were defined by CD45^dim^/SSC^low^ gating with additional backgating using CD34^−^ and CD117^−^ expression to improve the identification of the blast population when appropriate. For all markers, isotype controls were used to define the percentage of marker positive cells. In addition, the mean fluorescence intensity (MFI) ratio was calculated by dividing the geometric MFI of the antibody-stained sample by the geometric MFI of the respective isotype control. MFI ratio values > 1.5 were considered as significant [23]. MFI ratios as well as percent positive cells (PPCs) for each marker were compared between *TP53* aberrant AML and MDS samples using the Mann–Whitney *U* test. Multiple testing correction was performed using the Benjamini–Hochberg procedure to control the false discovery rate [24]. A *p* value below 0.05 was considered statistically significant. Principal component analysis (PCA) was performed on scaled MFI ratio and PPC data and visualized as a biplot of principal components 1 and 2, respectively, using the R package ggbiplot (v. 0.55) (https://github.com/vqv/ggbiplot). Samples with missing values were removed prior to PCA. Statistical analysis was performed with GraphPad Prism software version 9.1 (GraphPad Software) and R (v. 4.1.2).

## Results

A total of 84 patients with a median age of 71 years (range 34–86) suffering from myeloid malignancies with *TP53* aberrations were included, 46 of them with AML and 38 with MDS. The median observation period for the total cohort was 149 days (range, 5–2947). Demographic data of the patients are depicted in Table 1. Notably, de novo AML was the most frequent subtype in this disease category (60.8%), whereas therapy-related MDS accounted for the majority of MDS cases (42.0%). Transformation of MDS to AML was observed in 23 patients (60.5%) at a median time of 138 days (range, 30–2200) post diagnosis. With respect to laboratory parameters, platelet counts and LDH levels showed statistically significant differences between AML and MDS (*p* = 0.004 and *p* = 0.005, respectively, Mann–Whitney *U* test). Intensive chemotherapy treatments (“3 + 7” regimen) were more frequently given to AML patients, and allogeneic HSCT was performed in 5 AML (10.9%) and 3 MDS (8.1%) patients, respectively. In MDS patients, non-intensive therapies, preferably the hypomethylating drug azacitidine, were most commonly applied.

**Table 1:** Demographic data of study patients. Values are median (range) for continuous data or numbers (*n*, in %) for categorical data. *tAML* and *tMDS*, therapy-related AML and MDS; *sAML*, secondary AML; *MPN*, myeloproliferative neoplasm; *MLD*, multilineage dysplasia; *del(5q)*, deletion of 5q; *WBC*, white blood cells

Characteristics	AML, *n* = 46	MDS, *n* = 38	*p* value
Age (years)	70 (34–84)	72 (41–86)	0.242
Sex			
Female	24 (52.2%)	17 (44.7%)	0.519
*Disease classification*			
AML			
De novo	28 (60.8%)		
tAML	14 (30.4%)		
sAML post MPN	4 (8.6%)		
MDS			
MDS-EB		15 (39.3%)	
MDS with MLD		5 (13.1%)	
MDS with isolated del(5q)		2 (5.2%)	
tMDS		16 (42.0%)	
Transformation to AML		23 (60.5%)	
Prior malignancy	17 (36.9%)	17 (44.7%)	
*Laboratory values*			
WBC (G/L)	2.9 (0.8–123.7)	2.9 (1.1–23.5)	0.728
Bone marrow blasts (%)	55 (20–90)	10 (0–19)	< 0.001
Platelets (G/L)	44 (9–306)	85.5 (14–633)	0.004
Hemoglobin (g/dL)	8.8 (5.4–12.9)	8.9 (6.9–12.6)	0.411
Lactate dehydrogenase (U/L)	373 (114–1052)	252 (127–1412)	0.005
*Treatment*			0.015
Intensive chemotherapy	19 (41.4%)	5 (13.2%)	
Non-intensive therapy	17 (36.9%)	20 (52.6%)	
Best supportive care	10 (21.7%)	13 (34.2%)	

**Fig. 1:**
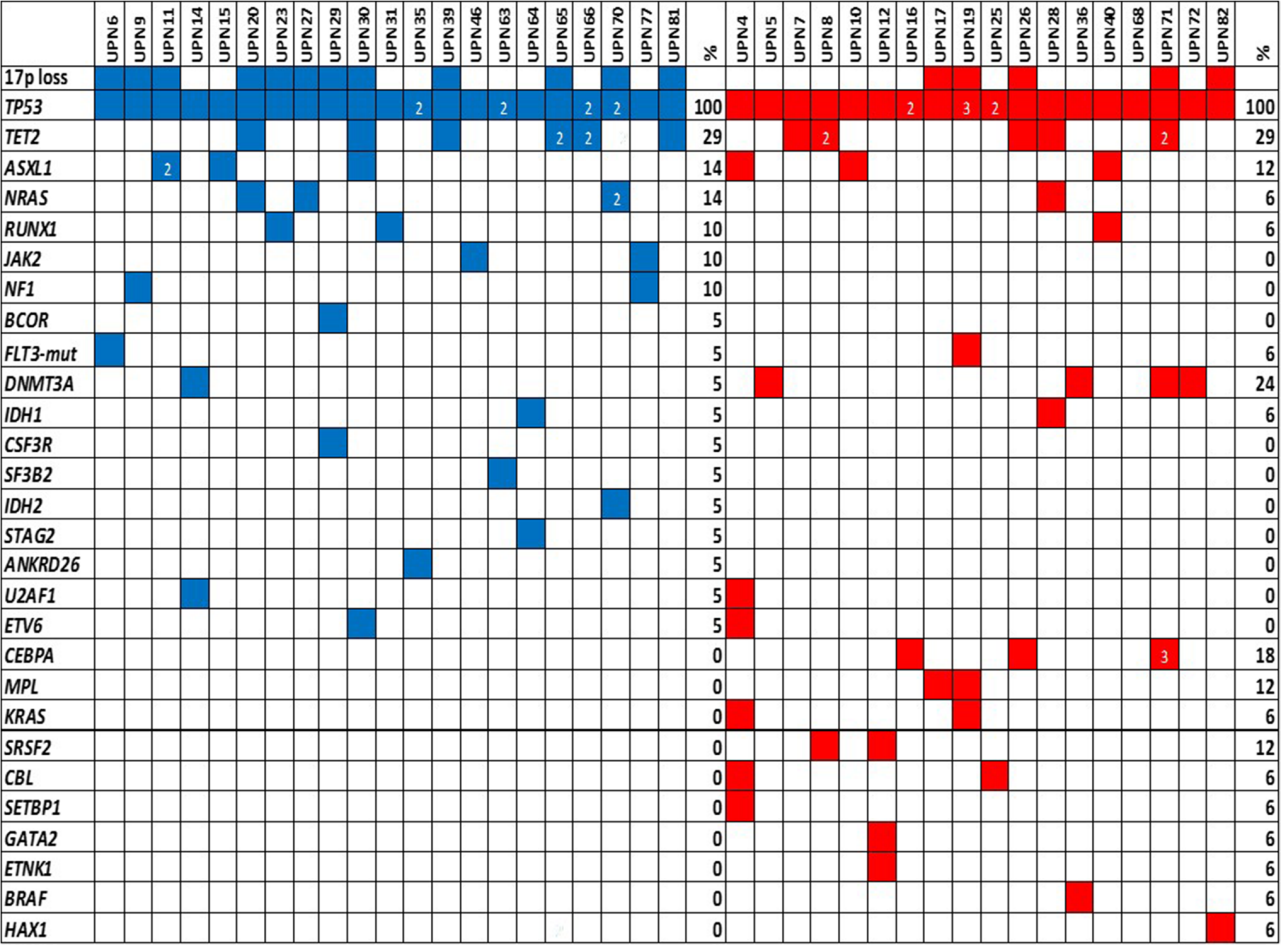
Cooperative mutations detected in patients with AML (blue) and MDS (red) with *TP53* aberrations. UPN, unique patient number; % refers to patients with a particular variant; numbers within boxes indicate multiple mutations within that gene

Cytogenetic and molecular genetic data including *TP53*-specific risk scores are shown in Table 2, Fig. 1, and Supplementary Table 2. Rates of complex karyotypes and deletions at 17p13.1, the locus of the *TP53* gene, were similar in both disease entities. Also, the number of patients with multiple *TP53* variants and the median *TP53* VAF was comparable between AML and MDS. The number of cooperating gene mutations as revealed by NGS of a panel of myeloid-associated genes was low in both groups. However, the genes affected differed between both groups with mutations in *KRAS*, *SRSF2*, *DNMT3A*, and *CEBPA* being more common in MDS. Due to the small number of cases, we refrained from statistical evaluation here.

**Table 2:** Genetic characteristics of diagnostic AML and MDS samples. Cytogenetic data are based on conventional karyograms and fluorescence in situ hybridization analyses. Values are median (range) for continuous data or numbers (*n*, in %) for categorical data. *VAF*, variant allele frequency

Characteristics	AML	MDS	*p* value
*Cytogenetics*			
Normal karyotype	3/40 (7.5%)	2/28 (7.1%)	1.000
Complex karyotype	36/40 (90.0%)	20/28 (71.4%)	0.060
Monosomy 17, deletion 17p13.1	19/40 (47.5%)	11/28 (39.3%)	0.621
*Molecular genetics*			
Patients with > 1 *TP53* variant	8/46 (17.4%)	10/38 (26.3%)	0.424
* TP53* VAF (%)	60 (8.9–98)	53.2 (4–91)	0.643
Availability of NGS data	31/46 (67.4%)	25/38 (65.8%)	
Patients with co-occurring mutations	21/32 (65.6%)	17/25 (68.0%)	1.000
No. of co-occurring mutations	1 (1–3)	2 (1–6)	0.379

**Fig. 2:**
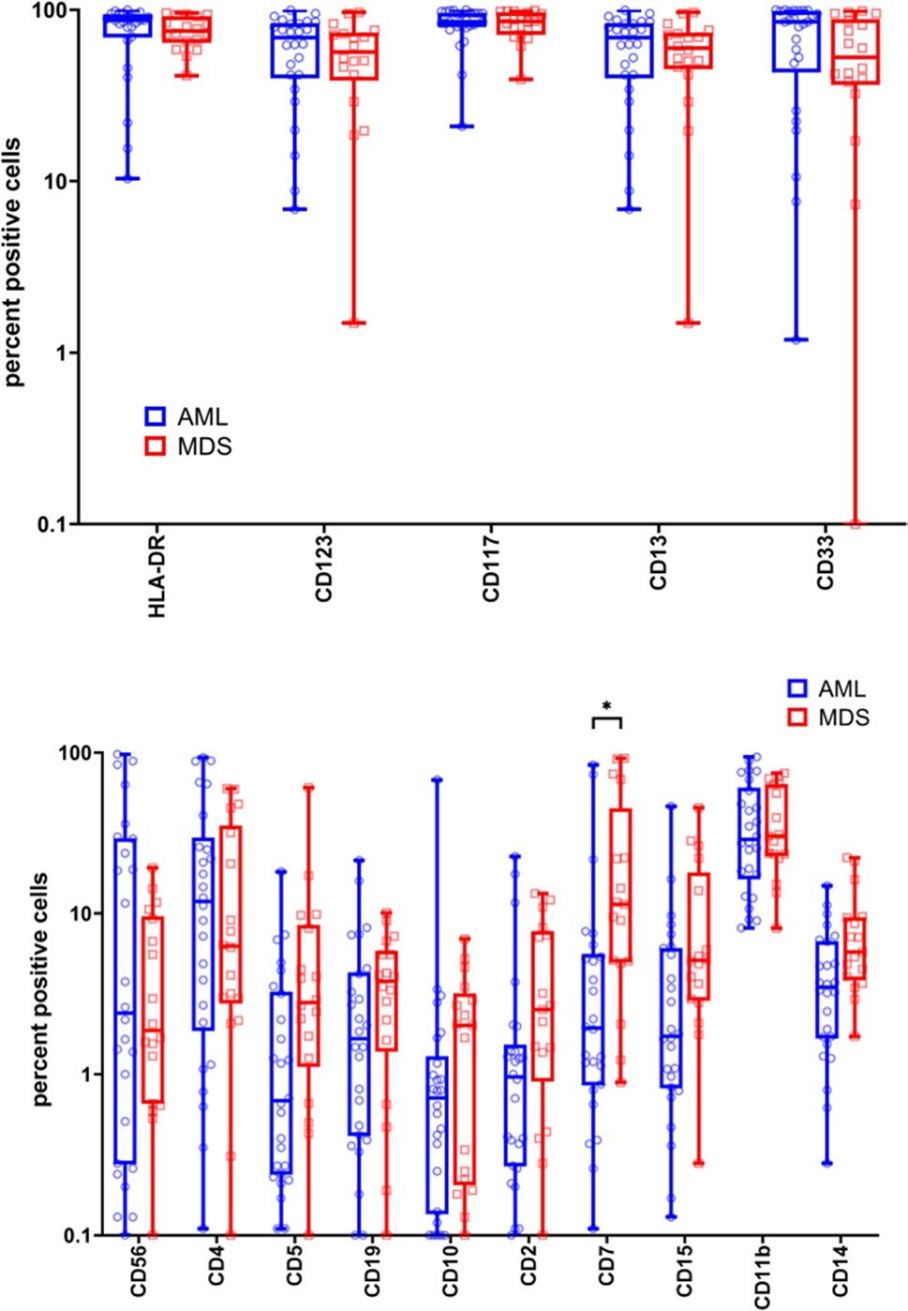
Percent positive cells expressing various aberrant (**A**) or myeloid progenitor markers (**B**) in AML (*n* = 26) and MDS samples (*n* = 18) as determined by flow cytometry analysis. Statistically significant differences in PPCs were only noted for CD7. *p < 0.05 as determined by the Mann–Whitney *U* test adjusted for multiple testing

**Fig. 3:**
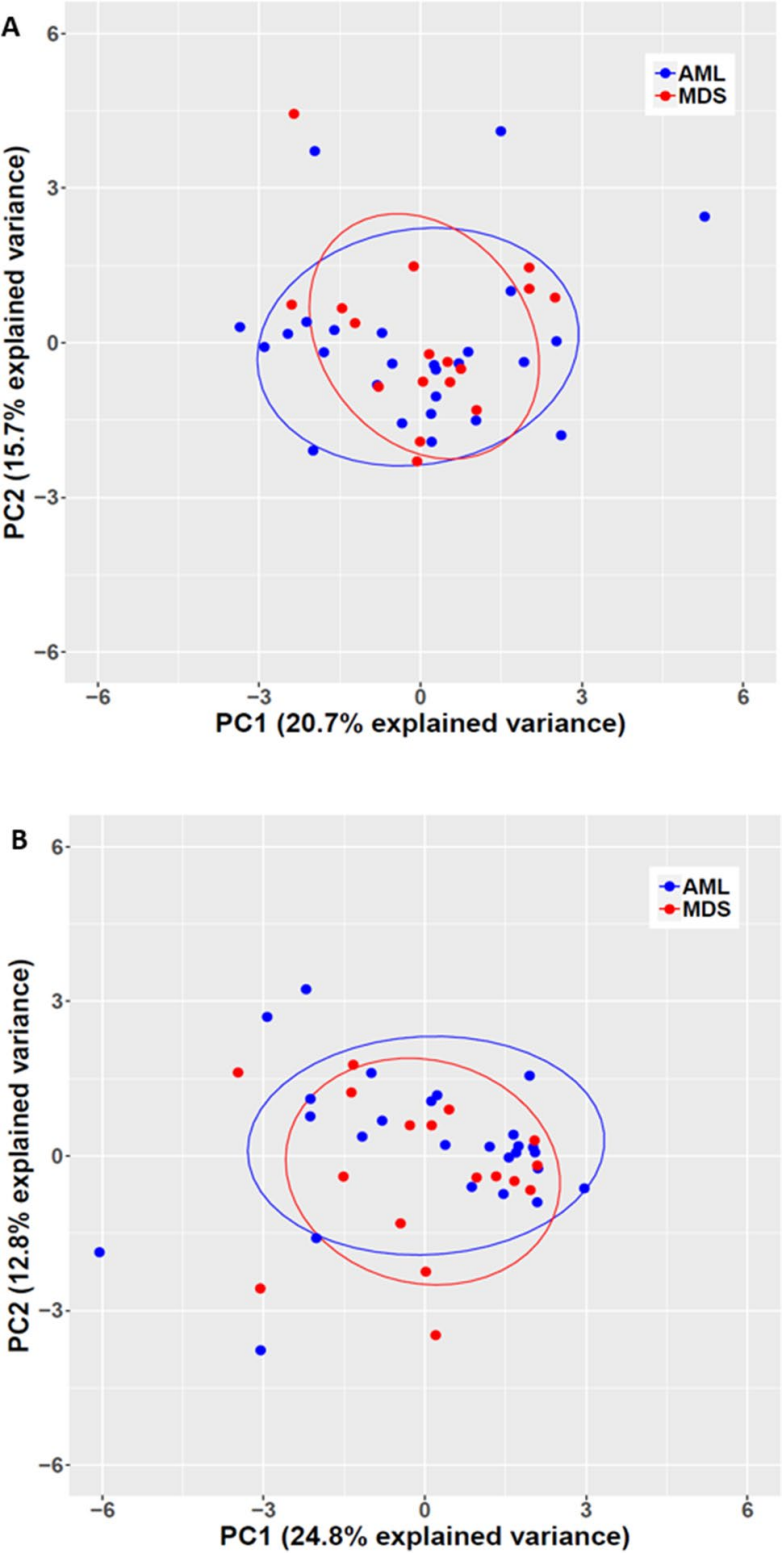
Principal component analysis (PCA) of immunophenotypic marker expression on immature blast cells of *TP53* aberrant AML (blue) and MDS (red) patients. **A** PCA of percent marker positive cells was used to evaluate the delineation of patients based on FACS analysis of 15 cell surface markers. AML and MDS samples cannot be separated by principal components 1 and 2, which explain 20.7% and 15.7% of the total variance, respectively. **B** PCA of the MFI ratio was used to evaluate the delineation of patients based on FACS analysis of 16 cell surface markers. AML and MDS samples cannot be separated by principal components 1 and 2, which explain 24.8% and 12.8% of the total variance, respectively. The points reflect the scores of the subjects; samples of a group are enclosed by a concentration ellipse with 68% probability

Immunophenotyping data obtained by multiparameter flow cytometry analysis at diagnosis were available in 26 AML and 18 MDS patients, respectively. By gating immature blast cells, we did not observe differences in the PPCs expressing typical markers of myeloid progenitor cells, such as CD13, CD33, CD117, CD123, and HLA-DR between diagnostic AML and MDS samples (Fig. 2A). Accordingly, expression levels of these markers as determined by MFI ratios did not differ, either (Supplementary Fig. 1). When analyzing aberrant marker expression on immature blast cells, we found significantly higher PPCs expressing CD7 in MDS as compared to AML samples. Other recurrently expressed aberrant markers, such as CD2, CD4, CD5, CD11b, CD14, CD15, CD19, and CD56, however, did not differ (Fig. 2B). Likewise, expression levels of aberrant markers were comparable in AML and MDS samples (Supplementary Fig. 2). In five patients, paired material at MDS diagnosis and AML progression was available. Myeloid as well as aberrant marker expression varied markedly between cases but were stable between disease stages in the individual subject (Supplementary Figs. 3 and 4). Finally, PCA of PPCs as well as of MFI ratios revealed that *TP53* aberrant AML and MDS samples could not be separated by immunophenotyping (Fig. 3).

**Fig. 4:**
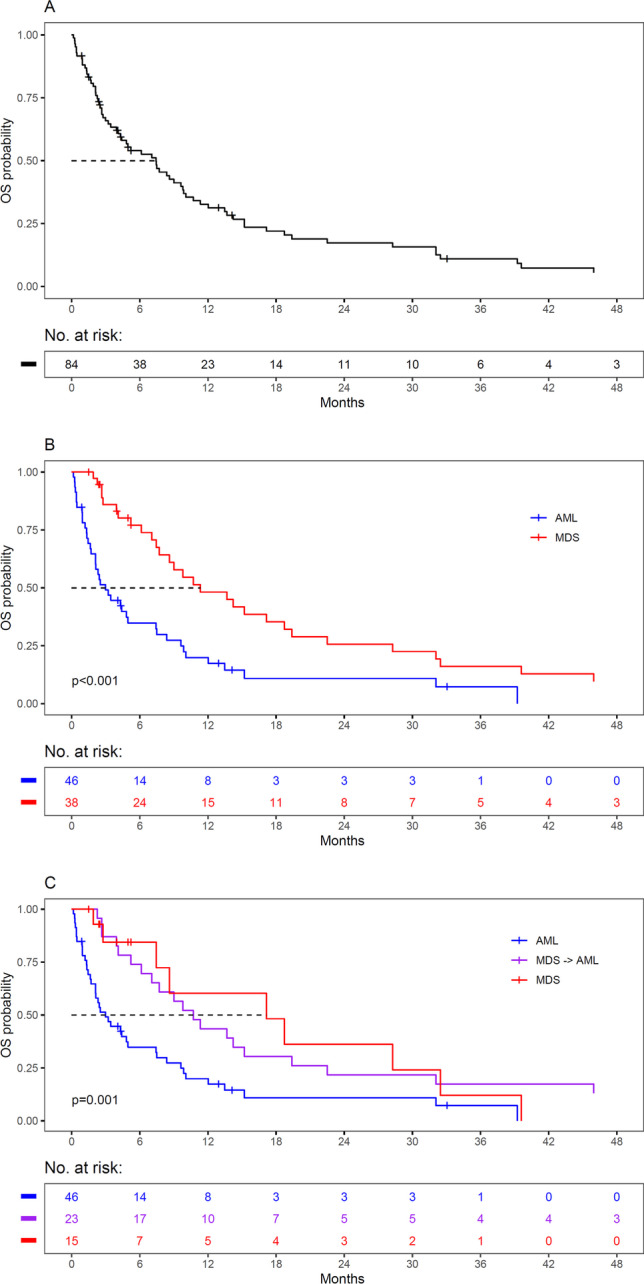
Survival of patients with AML and MDS with *TP53* aberrations. **A** OS probability of the total cohort of 84 patients. **B** Survival according to an initial diagnosis of AML or MDS. **C** Survival of AML, MDS, and transformed MDS patients, calculated from the time of MDS diagnosis

The median OS of the entire cohort was 226 days (95% CI, 131–300) (Fig. 4A) and was identical when patients were censored at allogeneic HSCT (95% CI, 131–298). Patients with MDS had a significantly better OS with a median of 345 days (95% CI, 235–590) as compared to those with AML with a median of 91 days (95% CI, 64–226) (Fig. 4B). Transformation of MDS to AML substantially reduced the prognosis of these patients (Fig. 4C). When calculated from the time of MDS diagnosis, the transformed group showed a median OS of 326 days (95% CI, 215–685), whereas the non-transformed group had one of 522 days (95% CI, 227–not reached). However, when calculated from the time of transformation, AML and transformed MDS patients had a similar median OS (Supplementary Fig. 5). The estimated 3-year OS rate was 11% for all patients (95% CI, 6–22), 7% for patients with AML (95% CI, 2–25), and 16% for those with MDS (95% CI, 7–36), respectively. With respect to treatment classes, patients with MDS showed superior survival as compared to those with AML throughout: intensive treatment including allogeneic HSCT, 1967.5 versus 255 days (*p* = 0.073); non-intensive treatment, 463 versus 98 days (*p* < 0.001); and best supportive care, 187 versus 32.5 days (*p* < 0.001).

Table 3 presents the results of univariable and multivariable Cox regression analyses assessing the impact of conventional risk parameters, the *TP53* VAF as well as the *TP53*-specific scores. In both uni- and multivariable analyses, the presence of AML and best supportive care as treatment as well as higher *TP53* VAFs were associated with a significantly shorter OS. However, neither of the *TP53*-specific risk scores reached statistical significance and were therefore not considered in the multivariable analysis.

**Table 3:** Univariable and multivariable Cox regression analyses for overall survival of AML and MDS patients with *TP53* aberrations. *HR*, hazard ratio; *CI*, confidence interval; *Ref.*, reference; *VAF*, variant allele frequency

		Univariable		Multivariable	
Variable	Category	HR (95% CI)	*p* value	HR (95% CI)	*p* value
Group	AML	1 (Ref.)			
	MDS	0.41 (0.25–0.67)	< 0.001		
Group (3 groups)	AML	1 (Ref.)			
	MDS– > AML	0.42 (0.24–0.73)	0.002	0.17 (0.07–0.42)	< 0.001
	MDS	0.39 (0.19–0.82)	0.012	0.17 (0.07–0.41)	< 0.001
Age		1.02 (0.99–1.04)	0.171	1.01 (0.97–1.05)	0.499
Sex	Female	1 (Ref.)		1 (Ref.)	
	Male	0.71 (0.44–1.14)	0.154	0.75 (0.39–1.45)	0.392
VAF		1.01 (1.00–1.03)	0.026	1.02 (1.01–1.03)	0.003
Disruptive/Non-disruptive	Disruptive	1 (Ref.)			
	Non-disruptive	1.68 (1.00–2.83)	0.050		
EAp53 score	< 75	1 (Ref.)			
	≥ 75	0.76 (0.44–1.29)	0.310		
Relative fitness score	≤ − 0.136	1 (Ref.)			
	> − 0.136	1.17 (0.57–2.39)	0.667		
Treatment class	Best supportive care	1 (Ref.)		1 (Ref.)	
	Intensive	0.34 (0.18–0.65)	0.001	0.08 (0.02–0.30)	< 0.001
	Non-intensive	0.50 (0.29–0.88)	0.016	0.24 (0.10–0.54)	0.001

## Discussion

Here, we present data comparing patients with *TP53* aberrant AML and MDS evaluated and treated at a tertiary cancer center. In agreement with our recently published postulation that they constitute a distinct disease entity [19], a multitude of features were, indeed, similar including clinical and cytogenetic parameters. In addition, the immunophenotype of immature blast cells did not differ significantly and a PCA revealed that *TP53* aberrant AML and MDS are undistinguishable by this approach. With a median OS of 226 days and an estimated 3-year survival rate of 11%, our study confirms previous results reporting an exceedingly adverse outcome of such cohorts [8, 9, 25, 26]. However, in our study, patients with MDS had a significantly better survival than those with AML independently of whether they were treated intensively or non-intensively or received best supportive care. It is well known that in AML and MDS, *TP53* mutations are early driver lesions affecting preleukemic/leukemic stem cells. As a sole event, *TP53* mutations are unable to induce neoplastic transformation as has been exemplified by their occurrence in healthy individuals with clonal hematopoiesis of indeterminate potential [27]. Secondary events are necessary for full transformation and in both, *TP53* mutated AML and MDS, preferably constitute chromosomal aberrations and copy number alterations leading to pronounced expression changes of co-operating gene [28]. Co-operating mutations have also been reported in *TP53* aberrant myeloid disorders although their frequency is substantially lower than in AML and MDS with a *TP53* wild-type status. In our NGS analysis, co-mutational patterns were different between *TP53* aberrant AML and MDS although the number of cases studied was too small to allow for an adequate statistical comparison. Several previous reports have unambiguously determined typical mutational patterns associated with MDS and secondary AML affecting genes like *SRSF2* and *SF3B1* [29–32]*.* We, therefore, speculate that the basis for manifestation as either *TP53* aberrant AML or MDS as well as their different survival probabilities is mainly due to different co-mutational events occurring secondary to the *TP53* aberration. In this regard, it would also be interesting to investigate molecular events responsible for transformation of *TP53* aberrant MDS to AML occurring within short periods of time.

The *TP53* VAFs were also comparable between AML and MDS. With a median value of 53% in MDS patients, it indicates that the mutant clone size is considerably exceeding the percentage of bone marrow blast cells in line with previous data on this issue [33]. Interestingly, the *TP53* VAF has been shown to be a statistically significant prognostic parameter in this study with higher values having a more pronounced adverse impact on survival. In a study of 1537 AML patients treated intensively within protocols of the AMLSG study group, we were able to demonstrate that even *TP53* VAFs of less than 20% represent significant adverse risk factors for both OS and event-free survival [26]. Furthermore, analysis of the same patient cohort revealed that the specific type of *TP53* mutation and its functional consequence has an impact on treatment response [21]. In contrast, in AML patients treated non-intensively with the hypomethylating drug azacitidine, an increasing mutant *TP53* load was associated with a significantly increased risk for treatment failure [34]. A systematic review and meta-analysis of studies of patients with MDS also reported a high *TP53* VAF as an independent prognostic parameter for survival with a 40% VAF as cutoff for high and low clonal burdens [35]. Recently, a report focusing on large cohorts of patients with MDS implemented the “*TP53* allelic state” as an important biological and clinical parameter. Ideally, the *TP53* allelic states should be assessed by combining conventional karyotyping + / − FISH and NGS analysis, respectively. Patients with *TP53* mono-allelic mutations were comparable to *TP53* wild-type patients, whereas multi-hit aberrations consistent with *TP53* bi-allelic events showed complex karyotypes and poor outcomes [36]. However, data on the value of the *TP53* allelic state in AML are not available yet.

Treatment of patients with *TP53* aberrant AML and MDS remains unsatisfactorily; however, novel promising drugs and strategies are being tested in several clinical trials. So far, the only curative approach is allogeneic HSCT indicating that restoration of intact immune surveillance mechanisms mediating graft-versus-leukemia takes a central role in combating the disease [18, 37]. Recently, a phase 3 trial evaluating venetoclax in combination with azacitidine showed superior OS rates in AML patients unfit for intensive therapy. In a subgroup analysis, CR rates for those with *TP53* mutations were 55% treated with that drug combination versus 0% in the azacitidine group. However, long-term survival did not improve and *TP53* mutations could be shown to be a major determinant of resistant disease [38–40]. Evaluating flotetuzumab, a bispecific CD3xCD123 antibody, and magrolimab, a CD47 antibody, together with azacitidine in patients with AML and MDS, respectively, revealed also high remission rates in *TP53* aberrant subgroups [41, 42]. APR-246 (eprenetapopt), a small molecule aiming at shifting mutant p53 towards a wild-type conformation, was recently tested together with azacitidine, in two phase 2 trials in patients with *TP53* mutated AML and MDS with encouraging CR and OS rates [43, 44]. A consecutive phase 3 study in patients with *TP53* mutant MDS failed to meet the primary endpoint of a significantly increased CR rate (Press Releases | Aprea Therapeutics). A comprehensive discussion of ongoing clinical trials in this particular patient cohort has recently been published [45].

In conclusion, our data on *TP53* aberrant AML and MDS, obtained at a tertiary cancer center, revealed a high concordance of biological and clinical features between these two disease entities. A significantly increased OS rate in MDS patients is likely due to a different co-mutational pattern. As *TP53* aberrations are early leukemogenic events in AML and MDS, these disease entities may, indeed, be classified and treated as a distinct disorder.

## Supplementary Information

Below is the link to the electronic supplementary material.

Supplementary file1 (PDF 487 KB)
