# Supplementary material for: Comparison of acute myeloid leukemia and myelodysplastic syndromes with TP53 aberrations
Source: Ann Hematol. 2022 Jan 26;101(4):837–46. doi: 10.1007/s00277-022-04766-2 (PMC8913568; doi:10.1007/s00277-022-04766-2)

Supplementary data

Comparison of acute myeloid leukemia and myelodysplastic syndromes with *TP53* aberrations

Sayantanee Dutta et al.

Supplementary Table 2. Antibodies used for immunophenotyping of blast cells of AML and MDS patients with *TP53* aberrations. Abbreviations: BD, Becton Dickinson; BC, Beckman Coulter.

Antibody	Fluorochome	Clone	Company
IgG1 (mouse)	FITC	X40	BD
IgG1 (mouse)	PE	X40	BD
IgG1 (mouse)	ECD	679.1Mc7	BC
IgG1 (mouse)	PC5.5	679.1Mc7	BC
IgG1 (mouse)	PC7	679.1Mc7	BC
IgG1 (mouse)	APC	679.1Mc7	BC
IgG1 (mouse)	APC-AF 700	679.1Mc7	BC
IgG1 (mouse)	APC-AF 750	679.1Mc7	BC
IgG1 (mouse)	PB	679.1Mc7	BC
IgG1 (mouse)	V500-C	X40	BD
HLA-DR	FITC	L243	BD
CD10	FITC	ALB1	BC
CD4	PE	13B8.2	BC
CD117	PE	104D2D1	BC
CD15	PE	HI98	BD
CD34	ECD	581	BC
CD123	PC5.5	SSDCLY107D2	BC
CD33	PC5.5	D3HL60.251	BC
CD19	PC7	J3-119	BC
CD13	PC7	Immu103.44	BC
CD11b	PC7	Bear1	BC
CD56	APC	N901	BC
CD2	APC	S5.2	BD
CD7	APC-AF 700	8H8.1	BC
CD5	APC-AF 750	BL1a	BC
CD14	APC-AF 750	RM052	BC
CD45	V500-C	2D1	BD

Supplementary Table 2. *TP53* mutations of patients with AML and MDS, analyzed in the study. Abbreviations: VAF, variant allele frequency; UPN, unique patient number; NA, not available. Disruptive, EAp53 and RFS refer to the *TP53*-specific scores as outlined in "Methods" of the main text.

Patient	*TP53* mutation	VAF	Disruptive	EAp53	RFS
UPN1	splice site mutation	85,3	NA	no score	no score
UPN2	p.R248Q;<br>p.V216M	3,7;<br>17,4	yes<br>no	78,95<br>73,3	-0,04<br>0,10
UPN3	p.C275Y	35,1	no	93,47	0,41
UPN4	p.L145P	11,2	no	72,75	0,23
UPN5	R248Q	48,8	yes	78,95	-0,04
UPN6	p.R156P	92	no	42,93	-0,62
UPN7	p.D281Y	79,5	no	96,23	0,36
UPN8	p.G245S	91	no	86,45	0,02
UPN9	p.N131I	62,6	no	81,75	0,30
UPN10	p.G262fs	51	yes	no score	no score
UPN11	p.V216M	66	no	73,3	0,10
UPN12	p.R273H	50	no	66,12	0,25
UPN13	p.R249T;<br>p.NK131K	36,8;<br>33,5	yes;<br>yes	95,93;<br>no score	0,51;<br>-0,01
UPN14	p.D281N	27,4	no	69,88	-0,002
UPN15	p.Q136E	60	no	50,94	-0,09
UPN16	p.D259fs;<br>p.V143M	30;<br>6	yes;<br>no	no score;<br>51,72	no score;<br>-1,33
UPN17	p.Y220C	77	no	72,52	0,09
UPN18	p.V216M;<br>p.C176R	25,7<br>7	no;<br>yes	73,3;<br>97,94	0,097;<br>0,39
UPN19	p.Q104X;<br>p.R273H;<br>p.Q65X	4,6;<br>24,3;<br>6,3	no;<br>no;<br>no	no score;<br>no score;<br>66,12	-0,29;<br>0,54;<br>no score
UPN20	p.K132R	77,5	no	69	0,46
UPN21	p.V216M	77,5	no	73,3	0,10
UPN22	p.E286*	37,5	yes	no score	0,85
UPN23	p.R156P	48,8	no	42,93	-0,62
UPN24	p.R248Q	72,5	yes	78,95	-0,04
UPN25	p.R175H;<br>splice site mutation	39;27;<br>40	no;<br>NA	78,51;<br>no score	-0,14;<br>no score
UPN26	p.R273H	68,2	no	66,12	0,25
UPN27	splice site mutation	77,0	NA	no score	no score
UPN28	p.H179L	37,3	yes	97,12	0,68
UPN29	p.E298*	61,8	yes	no score	no score
UPN30	p.C277G	49,4	no	93,39	-0,57

UPN31	splice site mutation	87,1	NA	no score	no score
UPN32	p.R280G	65	no	95,71	-0,52
UPN33	p.R175G	55,5	yes	95,2	0,54
UPN34	p.I255N	NA	no	87,39	-0,47
UPN35	p.R196G;<br>splice site mutation	14,6;<br>11,1	no	97,34;<br>no score	-1,88;<br>no score
UPN36	p.W146*	83	dis	no score	0,79
UPN37	splice site mutation	73,3	NA	no score	no score
UPN38	p.Y205C;<br>p. P278S	25,6	no;<br>no	77,88;<br>84,34	0,23;<br>0,16
UPN39	p.V216M	85,80	no	73,3	0,10
UPN40	P:R248Q	37,50	yes	78,95	-0,04
UPN41	p.E221fs	67,60	yes	no score	0,38
UPN42	p.R175H	NA	no	78,51	-0,14
UPN43	p.R175H	NA	no	78,51	-0,14
UPN44	p.Y234*	NA	yes	No score	0,18
UPN45	p.K132*;<br>p.R196P	NA	yes;<br>no	no score;<br>95,55	no score;<br>0,40
UPN46	p.R273C	NA	no	84,52	-0,13
UPN47	p.C238Y	NA	no	92,66	0,21
UPN48	p.T125P	NA	no	88,7	-0,18
UPN49	p.Y234H	NA	no	50,1	-0,07
UPN50	p.P278S	NA	no	84,34	0,17
UPN51	p.Asp228*	NA	yes	no score	no score
UPN52	p.Y220C	NA	no	72,52	0,22
UPN53	p.L114Pfs*56	NA	yes	no score	no score
UPN54	p.V173G	NA	yes	93,47	0,19
UPN55	p.R273H;<br>p.E286G	NA	no;<br>no	66,12;<br>93,06	0,25;<br>0,093
UPN56	p.Y220C	NA	no	72,52	0,22
UPN57	p.R273C	NA	no	84,52	-0,13
UPN58	p.Q165*,<br>p.L195P,	NA	yes;<br>yes	no score;<br>no score	-0,35;<br>0,39
UPN59	p.M237I;<br>p.P152L	NA	no;<br>no	63,68;<br>89,03	-1,19;<br>0,19
UPN60	p.G245S	Het	no	86,45	0,02
UPN61	p.I255T	57,0	no	70,87	-0,52
UPN62	p.R282W	60,5	no	73,21	0,02
UPN63	p.R248Q,<br>p.P27fs*50	14,3;<br>17	yes;<br>yes	78,95;<br>no score	-0,04;<br>no score
UPN64	p.P98A	21,5	no	89,49	no score
UPN65	p.R273H	81,3	no	66,12	0,25

UPN66	p.V274L;<br>p.R248W	50;<br>48,5	no,<br>yes	58,27;<br>84,11	-0,49;<br>0,07
UPN67	p.M237I	4	no	63,68	0,19
UPN68	p.H179N	98	yes	85,03	0,36
UPN69	p.V216L,<br>p.E349X	27,8;<br>20	yes;<br>no	71,23;<br>no score	0,097;<br>no score
UPN70	p.P250L ;<br>p.R273H	33,5;<br>50,8	no;<br>no	89;<br>66,2	-0.25;<br>0,25
UPN71	p. M237K	86,26	yes	85,52	0,30
UPN72	p. V157F	81	no	55,26	0,50
UPN73	p.R249M	NA	yes	95,41	0,17
UPN74	p.G245S	69	no	86,45	0,02
UPN75	p.K382fs*40	NA	yes	no score	no score
UPN76	p.R283fs*22	NA	yes	no score	no score
UPN77	p.H179R	50,1	no	81,91	0,22
UPN78	p.G245S	8,9	no	86,45	0,02
UPN79	p..R175H	65	no	78,51	-0,14
UPN80	p.G199E,	53,5	no	69,44	-1,97
UPN81	splice site mutation	69	NA	no score	no score
UPN82	p.I195T	77	yes	72,13	0,41
UPN83	p.R282G;<br>p.M246V	42;<br>42,5	no;<br>no	88,59;<br>66,21	0,46;<br>-0,72
UPN84	splice site mutation;<br>p.M246R	57;<br>23,5	NA;<br>yes	no score;<br>86,49	no score;<br>-1,17

Supplementary Figure 1. Geometric mean fluorescence intensity (MFI) of myeloid progenitor markers. No statistically significant difference in MFI values were noted between AML (n=26) and MDS (n=18) patients with *TP53* aberrations.

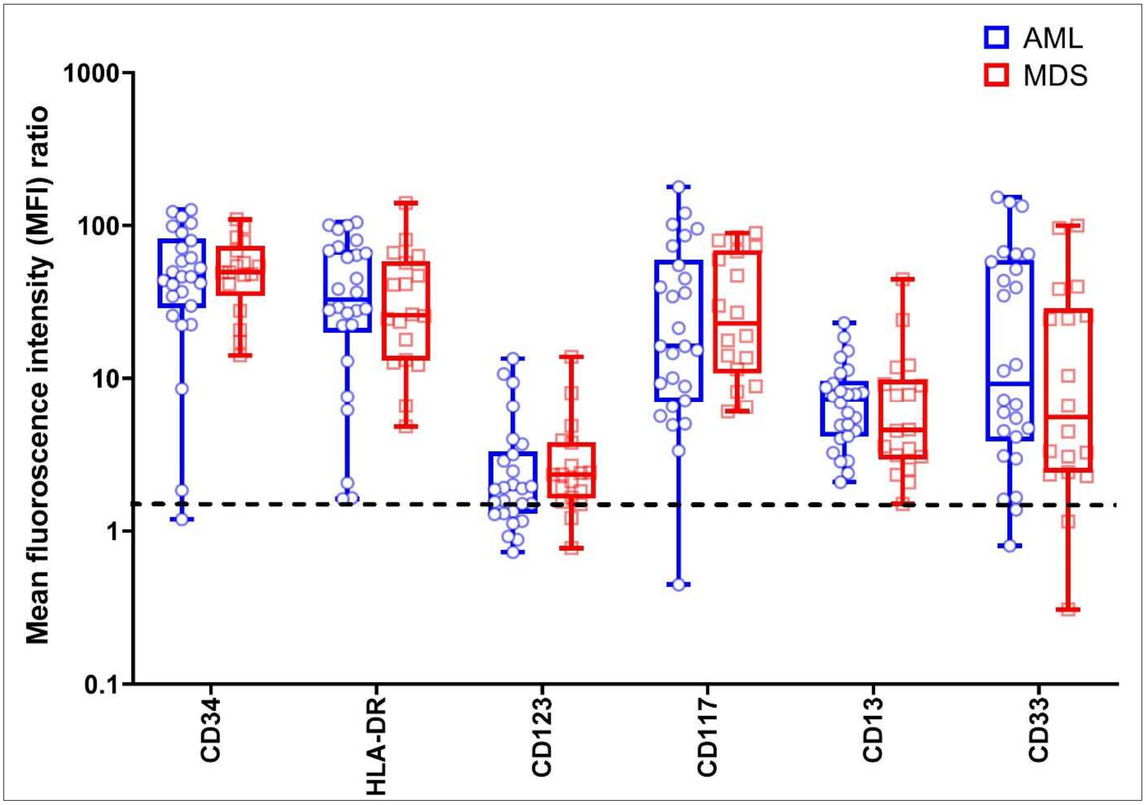

Supplementary Figure 2.Geometric mean fluorescence intensity (MFI) of aberrant markers. No statistically significant difference in MFI values were noted between AML (n=26) and MDS (n=18) patients with *TP53* aberrations.

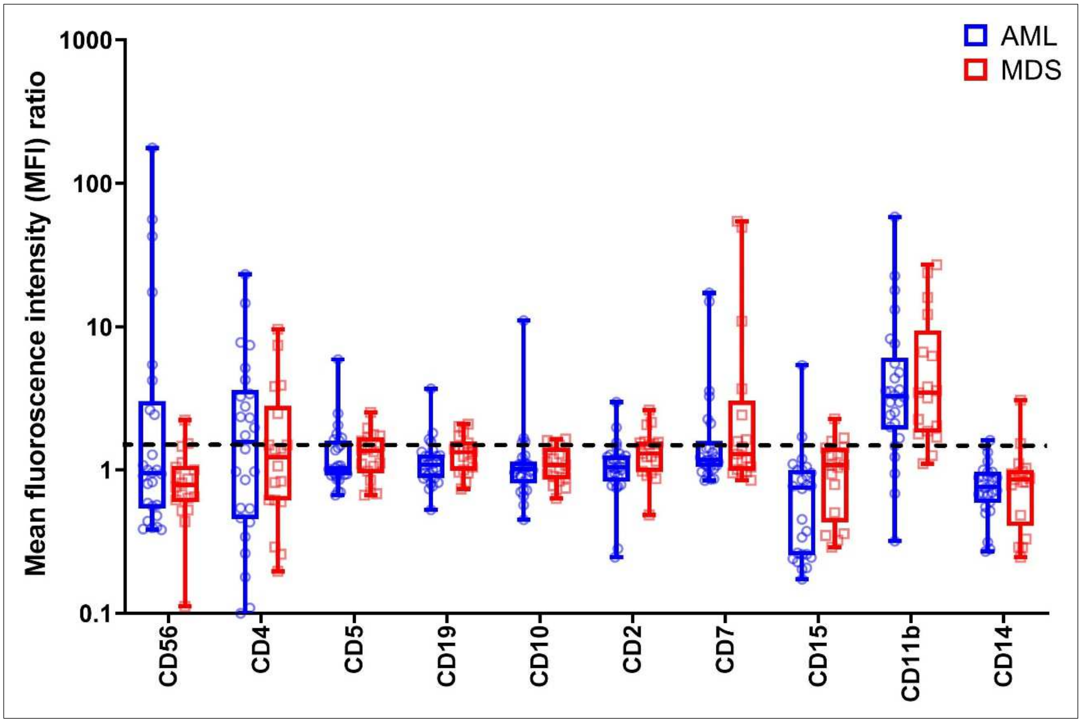

Supplementary Figure 3. Heatmap displaying percent (%) positive immature blast cells for surface markers of 5 MDS patients with *TP53* aberrations transformed to AML. Abbreviation: pat, patient.

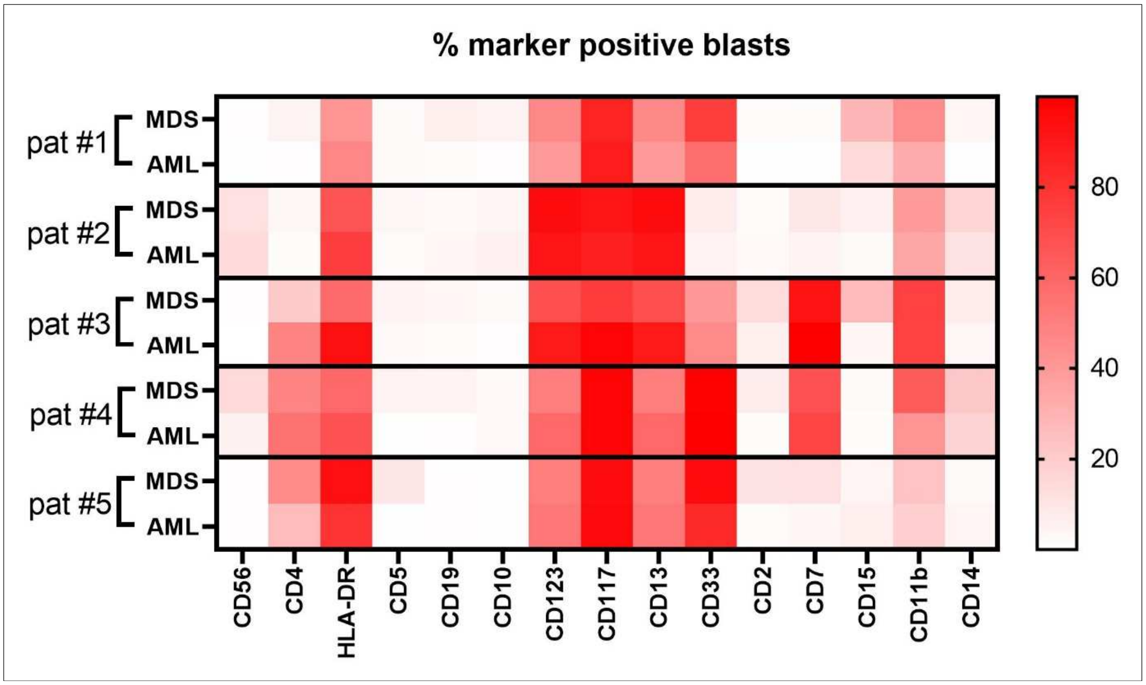

Supplementary Figure 4. Heatmap displaying the mean fluorescence intensity (MFI) for surface markers of 5 MDS patients with *TP53* aberrations transformed to AML. The MFI values are log10 transformed. Abbreviation: pat, patient.

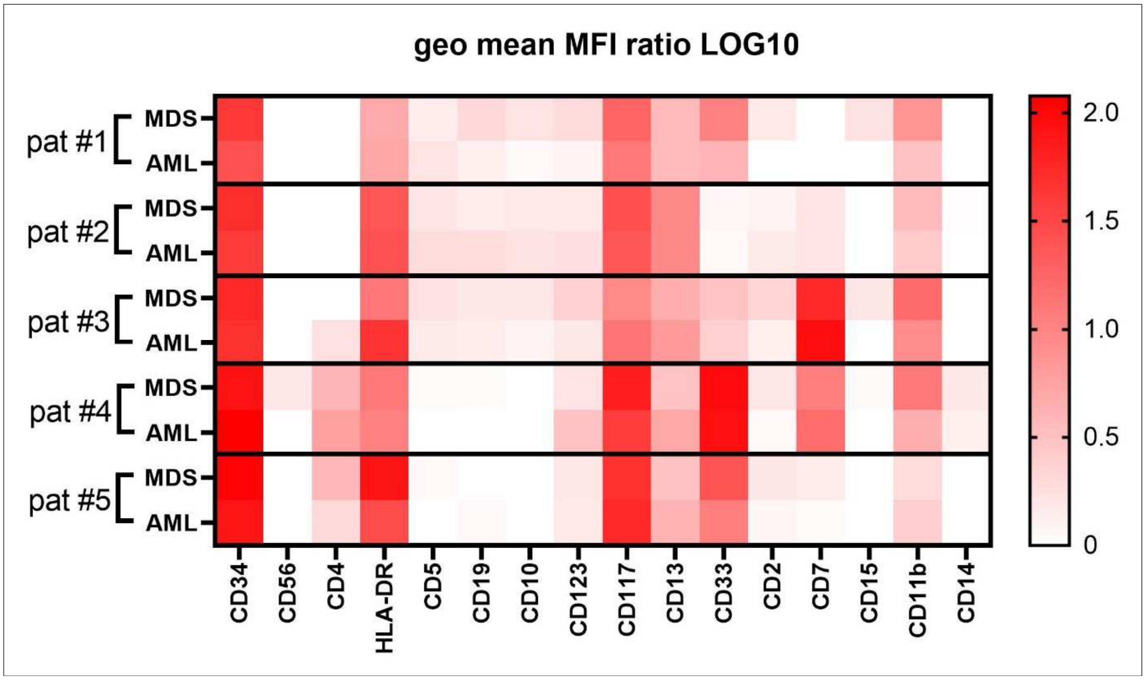

Supplementary Figure 5. Survival of patients with AML and MDS with *TP53* aberrations. Overall survival probability of AML and transformed MDS patients, calculated from the time of transformation.

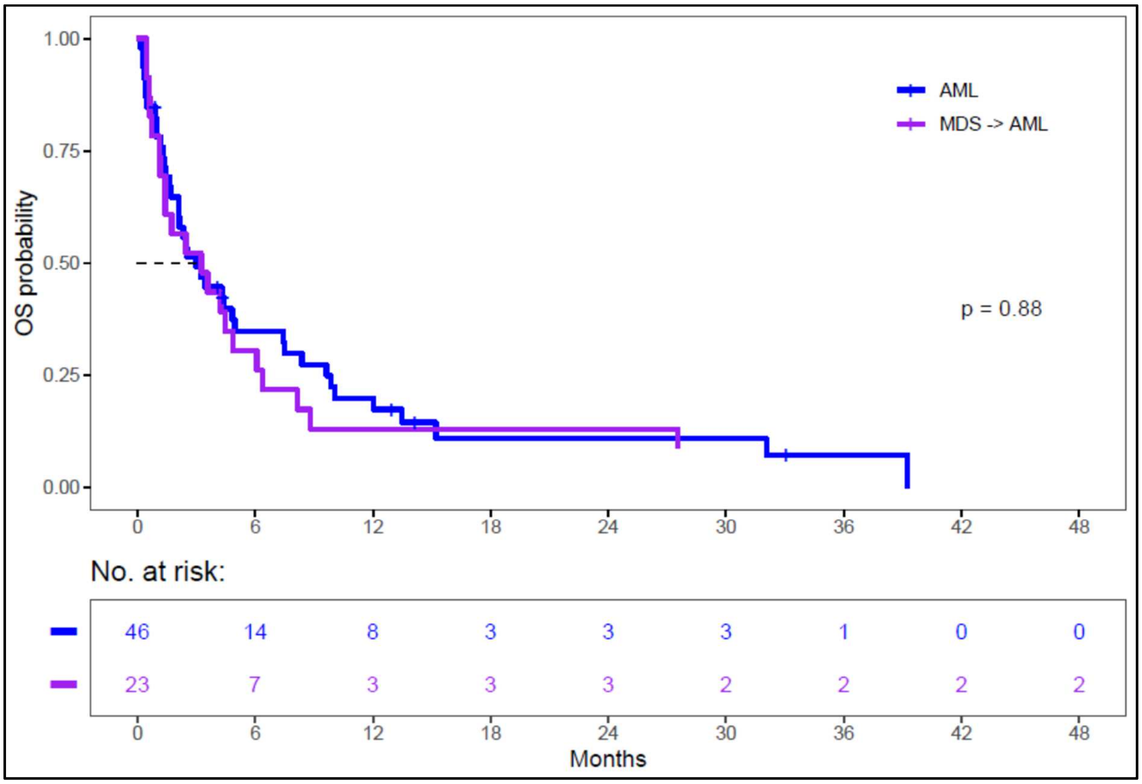

Supplement: Supplementary file 1 — Supplementary file1 (PDF 487 KB) [file 277_2022_4766_MOESM1_ESM.pdf]
